# Vaskulitische Beteiligung der Skelettmuskulatur und des peripheren Nervensystems: Klinische und neuropathologische Perspektive

**DOI:** 10.1007/s00393-024-01567-y

**Published:** 2024-09-24

**Authors:** Nikolas Ruffer, Felix Kleefeld, Marie-Therese Holzer, Martin Krusche, Ina Kötter, Udo Schneider, Werner Stenzel

**Affiliations:** 1 III. Medizinische Klinik und Poliklinik, Universitätsklinikum Hamburg-Eppendorf, Martinistraße 52, 20246 Hamburg, Deutschland; 2 Klinik für Neurologie, Charité – Universitätsmedizin Berlin, Berlin, Deutschland; 3 Klinik für Rheumatologie und Immunologie, Klinikum Bad Bramstedt, Bad Bramstedt, Deutschland; 4 Medizinische Klinik mit Schwerpunkt Rheumatologie und Klinische Immunologie, Charité – Universitätsmedizin, Berlin, Deutschland; 5 Institut für Neuropathologie, Charité – Universitätsmedizin, Berlin, Deutschland

**Keywords:** Vaskulitis, Myositis, Polyarteriitis nodosa, ANCA, Rheumatoide Arthritis, Vasculitis, Myositis, Polyarteritis nodosa, ANCA, Rheumatoid arthritis

## Abstract

Das periphere Nervensystem ist ein häufiges Zielorgan von systemischen Vaskulitiden. Daneben kann auch die Skelettmuskulatur betroffen sein. Myalgien, Paresen und Sensibilitätsstörungen sind in diesem Zusammenhang typische Krankheitszeichen, die zu schwerwiegenden Funktionseinschränkungen und einer Beeinträchtigung der Lebensqualität führen können. Eine vaskulitische Affektion der Skelettmuskulatur (vaskulitische Myopathie, VM) und peripherer Nerven (vaskulitische Neuropathie, VN) tritt vorwiegend bei der Polyarteriitis nodosa und Kleingefäßvaskulitiden auf. Die VM präsentiert sich mit erhöhten Entzündungsparametern und ist typischerweise durch immobilisierende Myalgien mit normwertiger Kreatinkinaseaktivität und diffuse oder fleckige Hyperintensitäten in der T2-Wichtung in der MRT-Bildgebung gekennzeichnet („MRT-Myositis ohne Myositis“). Bei der VN entwickeln sich vorwiegend im Bereich der unteren Extremität sensomotorische Defizite im Versorgungsgebiet mehrerer peripherer Nerven (z. B. Mononeuritis multiplex) mit akuter bis subakuter Anamnese. Die histopathologische Untersuchung von Nerven- und Muskelbiopsien ist der Goldstandard für die Diagnose vaskulitischer Manifestationen und hat einen bedeutsamen Einfluss auf das therapeutische Vorgehen.

## Abstract (en)

The peripheral nervous system is a classic target organ in systemic vasculitis. In addition, the skeletal muscle can also be affected. Myalgia, muscle weakness and sensory deficits are typical signs, which can lead to severe functional limitations and impaired of quality of life. Vasculitic involvement of the skeletal muscle (vasculitic myopathy [VM]) and peripheral nerves (vasculitic neuropathy [VN]) occurs predominantly in polyarteritis nodosa and small-vessel vasculitis. VM presents with elevated markers of inflammation and is typically characterized by immobilizing myalgia with normal creatine kinase activity and diffuse or patchy areas of hyperintensity on T2-weighted MRI (“MRI myositis without myositis”). In VN, sensor motor deficits predominantly affect the lower extremity in the area supplied by several peripheral nerves (e.g., mononeuritis multiplex) with acute to subacute history. The histopathological examination of nerve and muscle biopsies is the gold standard for the diagnosis of vasculitic manifestations and has a significant impact on the therapeutic approach.

## Einleitung

Die morphologische Diagnose einer Vaskulitis beruht auf dem Nachweis von Entzündungszellinfiltraten innerhalb der Gefäßwand bzw. perivaskulären Infiltraten sowie begleitenden Befunden einer Gefäßwandschädigung [1]. Hierbei können insbesondere Gefäßwandnekrosen und eine Leukozytoklasie bestehen [1]. Ergänzend können extravaskuläre Entzündungszeichen (z. B. granulomatöse Veränderungen) auftreten [1].

Ätiologisch werden systemische (primäre) Vaskulitiden (SV), sekundäre Vaskulitiden (im Rahmen bestimmter Grunderkrankungen) und Einzelorganvaskulitiden (single-organ vasculitis, SOV) unterschieden [1]. In der Nomenklatur der Vaskulitiden (2012 Chapel Hill Consensus Conference) werden die primären Vaskulitiden wiederum in drei Hauptkategorien anhand des vorwiegenden Befallsmusters eingeteilt [2]: Großgefäßvaskulitiden, Vaskulitiden mittelgroßer Gefäße und Kleingefäßvaskulitiden. Allerdings können alle Vaskulitisentitäten der genannten Kategorien Arterien jedes Gefäßkalibers befallen [2]. Beispielsweise können sich auch Großgefäßvaskulitiden mit dem Befall kleiner Arterien manifestieren [2].

Im Allgemeinen wird das klinische Bild eines Vaskulitissyndroms wesentlich vom (vorwiegenden) Gefäßbefallsmuster bestimmt. Während der Befund einer palpablen Purpura auf histopathologischer Ebene dem Befall von Kleingefäßen entspricht, kann eine Nierenarterienstenose oder mesenteriale Ischämie der Ausdruck einer Großgefäßvaskulitis sein. Der histologische Entzündungstyp, das Organbefallsmuster (z. B. pulmorenales Syndrom) und die Autoimmunserologie geben zusätzliche Anhaltspunkte für die klinische Einordnung und letztendliche Diagnosestellung.

**Abb. 1:**
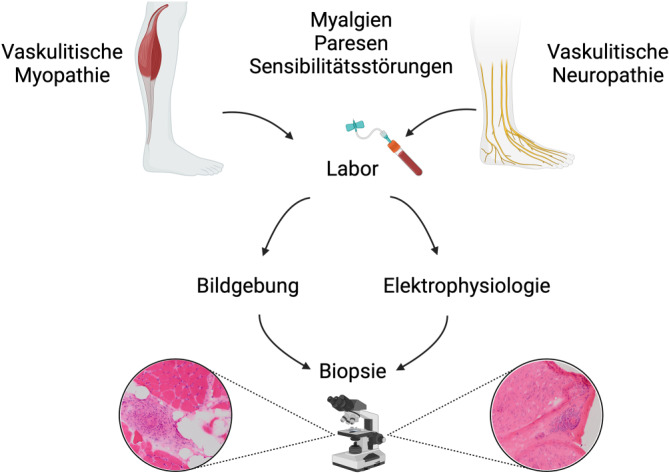
Die Diagnose der vaskulitischen Myopathie bzw. Neuropathie beruht auf der Integration von klinischen, laboranalytischen, apparativen und histopathologischen Befunden. Neuromuskuläre Symptome, die von Allgemeinsymptomen (z. B. Fieber) oder klinischen Surrogatparametern (z. B. palpable Purpura, subkutane Noduli, alveoläre Hämorrhagie) für ein Vaskulitissyndrom begleitet werden, lenken den Verdacht auf eine entzündliche Systemerkrankung. Mithilfe der Labordiagnostik können die systemischen Entzündungszeichen belegt und Differenzialdiagnosen evaluiert werden. Basierend auf Bildgebung und Elektrophysiologie muss die Indikation zur Muskel‑/Nervenbiopsie evaluiert werden, die eine Diagnosesicherung erlaubt. Abbildung erstellt mit BioRender.com

Myalgien, Paresen und Sensibilitätsstörungen sind häufige Krankheitszeichen von systemischen Vaskulitiden mit Befall des peripheren Nervensystems (PNS) und der Skelettmuskulatur [3–7]. Eine direkte vaskulitische Affektion der Skelettmuskulatur und peripherer Nerven tritt hauptsächlich bei der Polyarteriitis nodosa (PAN) und Kleingefäßvaskulitiden auf (Tab. 1). Die Diagnosesicherung beruht hierbei auf der Integration von klinischen, laboranalytischen, apparativen und histopathologischen Befunden (Abb. 1; Tab. 2). Idealerweise erfolgt eine interdisziplinäre Falldiskussion von Kliniker:innen und Neuropatholog:innen. Beispielsweise ist die vaskulitische Myopathie (VM) nicht mit einer Myositis gleichzusetzen und die sichere Unterscheidung der beiden Entitäten ist gegenwärtig nur durch Muskelbiopsie möglich [8]. Die Interpretation von T2-Hyperintensitäten in der Magnetresonanztomografie (MRT) als „Myositis“ kann in diesem Zusammenhang irreführend sein [8].

**Tab. 1:** Übersicht der Häufigkeiten neuromuskulärer Manifestationen bei systemischen Vaskulitiden mit vorwiegender Beteiligung der Skelettmuskulatur und des peripheren Nervensystems

Vaskulitis	Myalgien	Myopathie	Neuropathie
Polyarteriitis nodosa	Bis zu 50 %	Unbekannt, Fallserien	Bis zu 50 %
ANCA-assoziierte Vaskulitis	Bis zu 25 %	4,2–28,2 %	GPA/MPA 10–25 % EGPA bis zu 80 %
Kryoglobulinämische Vaskulitis	Unbekannt, typisches Symptom im Schub	Unbekannt, Einzelfallberichte	17 bis 80 %
Rheumatoide Vaskulitis	Unbekannt, typisches Symptom im Schub	Unbekannt, Fallserien	35 bis 63 %

Abkürzungen: *ANCA* antineutrophile zytoplasmatische Antikörper, *EGPA* eosinophile Granulomatose mit Polyangiitis, *GPA* Granulomatose mit Polyangiitis, *MPA* mikroskopische Polyangiitis

**Tab. 2:** Krankheitszeichen und Diagnostik bei vaskulitischer Neuropathie und Myopathie

	Vaskulitische Myopathie	Vaskulitische Neuropathie
*Symptome*	Distal betonte Myalgien (typischerweise untere Extremität)	Schmerzhafte Parästhesien
*Klinische Befunde*	Paresen	Paresen
		Hypästhesie, Anästhesie
		Mononeuritis multiplex
		Distal symmetrische Polyneuropathie
	Fieber, Gewichtsverlust	
	Hautvaskulitis	
	Glomerulonephritis	
	Alveoläre Hämorrhagie	
	Myokardiale Inflammation	
	Erosive Polyarthritis	
*Labor*	Differenzialblutbild	
	C‑reaktives Protein, Blutsenkungsgeschwindigkeit	
	Kreatinin, Kreatinkinase, Urindiagnostik	
	ANCA, Anti-CCP-Ak, RF, ANA	
	Komplement C3/C4, Kryoglobuline	
	Virusserologien (HBV, HCV, HIV)	
	Ggf. Myositis spezifische/assoziierte Antikörper	Ggf. Neurofilament-Leichtketten
*Apparative Diagnostik*	Magnetresonanztomografie	Elektrophysiologische Diagnostik
	Ggf. Sonografie	
*Muskel‑/Nervenbiopsie*	Hämatoxylin-Eosin, Gömöri-Trichrom, Elastica-van-Gieson, Eisen CD3, CD20, CD45, CD68	

Abkürzungen: *ANA* antinukleäre Antikörper, *ANCA* antineutrophile zytoplasmatische Antikörper, *Anti-CCP-Ak* Antikörper gegen zyklische citrullinierte Peptide, *HBV* Hepatitis-B-Virus, *HCV* Hepatitis-C-Virus, *HIV* humanes Immundefizienz-Virus, *RF* Rheumafaktor

Die Biopsieentnahme und histopathologische Begutachtung von Nerven- und Muskelbiopsien ermöglicht eine sichere Diagnosestellung und hat einen bedeutsamen Einfluss auf das therapeutische Vorgehen (z. B. Immunsuppression mit Cyclophosphamid [CYC] bei organbedrohender Manifestation durch eine Mononeuritis multiplex [MM]).

## Systemische Vaskulitiden

### Polyarteriitis nodosa.

Die PAN ist eine nekrotisierende Vaskulitis mit vorwiegendem Befall mittelgroßer Arterien, die nicht mit antineutrophilen zytoplasmatischen Antikörpern (ANCA) assoziiert ist [2]. Der häufig beschriebene Zusammenhang mit einer Hepatitis-B-Virusinfektion spielt in Europa heutzutage keine Rolle mehr [7]. Stattdessen tritt die sekundäre PAN häufig im Rahmen von myelodysplastischen Syndromen, soliden Tumoren oder dem familiären Mittelmeerfieber auf [7]. Typischerweise sind die Haut (z. B. Livedo racemosa), das periphere Nervensystem (z. B. MM), die Nieren (z. B. renale Mikroaneurysmata) und der Gastrointestinaltrakt (z. B. mesenteriale Ischämie) betroffen [4, 6, 7]. Begleitend treten Allgemeinsymptome wie Fieber und Gewichtsverlust auf. In bis zu 50 % der Fälle entwickeln sich Myalgien [4, 6, 7]. Klinische oder histopathologische Befunde einer Kapillaritis (z. B. Glomerulonephritis) oder eine Affektion des venösen Systems schließen die Diagnose einer PAN aus [9]. Kohortenstudien mit histologischen Untersuchungen zur Häufigkeit einer VM bei PAN liegen nicht vor. Einzelfallberichte und Fallserien beschreiben einen Subtyp mit vorwiegendem Befall der distalen Muskulatur der unteren Extremität und serologischer Inflammation („muskuläre PAN“) [10, 11]. Zusätzlich scheint ein Befall der Skelettmuskulatur bei der PAN mit einem charakteristischem Befund in der ^18^FDG-PET/CT assoziiert zu sein („dirty muscle sign“) [12, 13]. Ferner kann eine Biopsie des Musculus gastrocnemius die diagnostische Ausbeute auch bei normwertiger Kreatinkinase (CK) und fehlender Klinik wesentlich erhöhen, falls die Verdachtsdiagnose anderweitig nicht gesichert werden kann [9]. Klinische Zeichen einer peripheren Neuropathie treten bei etwa 50 % der Erkrankten auf, am häufigsten als MM [4, 6, 7]. Bei dieser kommt es zu sensiblen und/oder motorischen Ausfällen im Versorgungsgebiet mehrerer peripherer Nerven. Neuropathische Schmerzen und begleitende sensible Defizite mit kurzer Anamnese sind in Abwesenheit von Risikofaktoren für eine Polyneuropathie (PNP) hochverdächtig auf eine PAN [9]. Die kombinierte Biopsie von Muskel und Nerv kann hier wegweisend sein (z. B. Nervus peroneus superficialis und Musculus peroneus brevis; Nervus suralis und Musculus gastrocnemius) [14, 15].

### ANCA-assoziierte Vaskulitiden (AAV).

Basierend auf der gegenwärtigen Nomenklatur werden drei Entitäten unterschieden [2]: Granulomatose mit Polyangiitis (GPA), mikroskopische Polyangiitis (MPA) und eosinophile Granulomatose mit Polyangiitis (EGPA). Es handelt sich um Kleingefäßvaskulitiden, die bevorzugt das periphere Nervensystem, die Lunge sowie die Nieren befallen [5]. Der ANCA-Status^1^ (Proteinase 3, PR3-ANCA; Myeloperoxidase, MPO-ANCA), das Differenzialblutbild sowie histopathologische und radiologische Befunde unterstützen die klinische Einordnung. Obwohl die Organbefallsmuster und einzelne Krankheitsmanifestationen vielfältige Überschneidungen aufweisen, lassen sich typische Befundkonstellationen identifizieren. Während eine pulmonale Beteiligung in Form einer interstitiellen Lungenerkrankung (usual interstitial pneumonia) fast ausschließlich bei MPO-ANCA-assoziierter Vaskulitis auftritt, finden sich pulmonale Noduli mit Kavernenbildung praktisch nur bei PR3-ANCA-assoziierter Vaskulitis [5]. Bei der EGPA können MPO-ANCA in bis zu 30 % der Fälle nachgewiesen werden und sind verstärkt mit der Entwicklung vaskulitischer Manifestationen (Glomerulonephritis, periphere Neuropathie) assoziiert [16]. Muskuloskelettale Beschwerden werden von bis zu 80 % der Erkrankten mit AAV berichtet [17–19]. Myalgien werden wiederum in etwa einem Viertel aller Fälle angegeben [5]. Die diagnostische Ausbeute einer Muskelbiopsie bei AAV lag in einer retrospektiven Studie [20] bei 58 %, die Prävalenz einer VM betrug 16,3 % in der Gesamtkohorte. Myalgien lagen nur bei der Hälfte der Patient:innen mit positiver Biopsie vor. Erhöhte CK-Werte fanden sich nur in 10 % aller Fälle mit Nachweis einer VM, hierbei handelte es sich in allen Fällen um Erkrankte mit EGPA [20]. Ein positiver Befund in der Muskelbiopsie war hier mit weiblichem Geschlecht, MPO-ANCA und Neutrophilie assoziiert [20]. In einer weiteren Kohortenstudie [19] wurde die Häufigkeit einer VM bei der MPA mit 4,2 % angegeben. Eine systematische Übersichtsarbeit zur Muskelbeteiligung bei systemischen Vaskulitiden fand die meisten Fallberichte bei der MPA [3]. Die Häufigkeit einer peripheren Neuropathie wird wesentlich durch den ANCA-Status und die Grunderkrankung bestimmt [5, 21]. Die EGPA zeigt hierbei die stärkste Tendenz für den Befall des peripheren Nervensystems, bei Nachweis von MPO-ANCA sind sogar bis 80 % der Erkrankten betroffen [16]. Bei der GPA und MPA entwickelt sich eine periphere Neuropathie in etwa 10–25 % der Fälle [17–19]. Aus klinischer Sicht steht der Befall motorischer Nerven im Vordergrund, begleitende lebensbedrohliche Organmanifestationen treten jedoch nicht gehäuft auf [22].

### Kryoglobulinämische Vaskulitis (KV).

Die KV ist eine Immunkomplex-Vaskulitis, die hauptsächlich Kleingefäße betrifft [23]. Kryoglobuline sind zirkulierende Immunglobuline, die in vitro spontan bei Temperaturen unter 37 °C ausfallen und mit Endorganschäden assoziiert sind. Die häufigste Ursache einer Kryoglobulinämie ist die Hepatitis-C-Virusinfektion. Weitere Ursachen sind Autoimmunerkrankungen und lymphoproliferative B‑Zell-Erkrankungen [24]. Klinisch besteht eine vorwiegende Affektion der Haut, der Niere und des peripheren Nervensystems. Begleitend können Allgemeinsymptome wie Abgeschlagenheit, Müdigkeit und Fieber auftreten. In der klinisch-neurologischen Untersuchung zeigt sich am häufigsten eine distal symmetrische sensorische (PNP) [25]. Motorische Symptome entwickeln sich teilweise erst nach Monaten oder Jahren, können aber auch komplett ausbleiben. Deutlich seltener besteht hingegen eine MM (etwa ein Drittel der Fälle), bei der häufig der Nervus peroneus betroffen ist [23]. Sehr wenige Fallberichte beschreiben eine VM [3].

### Rheumatoide Vaskulitis (RV).

Die RV ist eine seltene extraartikuläre Manifestation der rheumatoiden Arthritis (RA) [26]. Es sind hauptsächlich Erkrankte mit langjährigem und erosivem Krankheitsverlauf („ausgebrannte RA“) sowie persistierender Krankheitsaktivität betroffen [26, 27]. Weitere Risikofaktoren sind männliches Geschlecht, Nikotinkonsum, Seropositivität (Rheumafaktor, RF; Anti-CCP-Antikörper) und zusätzliche extraartikuläre Manifestationen [26, 27]. Typische Organmanifestationen betreffen die Haut (z. B. Bywater-Läsionen, Livedo racemosa, scharf begrenzte Ulzerationen der Unterschenkel), das periphere Nervensystem (z. B. MM, distal symmetrische sensible PNP), das Herz (z. B. Perikarditis) und das Auge (z. B. Skleritis) [26, 27]. Am häufigsten entwickelt sich eine MM (etwa 50 % der Fälle), bei der fast immer der Nervus peroneus betroffen ist [28]. Die Neuropathie geht meistens mit einer Beteiligung intramuskulärer Gefäße (bis zu 86 % der Fälle) einher [29]. Es bestehen klinische (Organbefallsmuster) und histopathologische (vorwiegender Befall mittelgroßer Arterien) Überschneidungen zwischen der RV und der PAN [3, 26, 27]. Zusätzlich kann die RV auch als Kleingefäßvaskulitis mit Muskelbeteiligung auftreten. Der Nachweis von perivaskulären Infiltraten (≥ 3 Zellschichten) in Muskelbiopsien bei RA^2^ scheint mit dem Vorliegen einer RV zusammenzuhängen [31]. Der Goldstandard^3^ für die Diagnose einer RV basiert auf dem histopathologischen Nachweis von vaskulitischen Veränderungen im Kontext einer RA und kann mithilfe von Hautbiopsien (tiefe Hautbiopsie vom Ulkusrand) oder einer kombinierten Muskel-Nerven-Biopsie diagnostiziert werden [15, 26, 27]. Bei entsprechender Anamnese sollte eine medikamentös-induzierte Kleingefäßvaskulitis durch TNF-α-Antagonisten als Differenzialdiagnose bedacht werden [26, 27].

## Kollagenosen mit sekundärer Vaskulitis

Eine sekundäre Vaskulitis mit Neuropathie kann auch beim primären Sjögren-Syndrom (SjS) auftreten [32]. In bis zu 50 % der Fälle besteht auch eine gemischte Kryoglobulinämie (Typ II/III), die über eine Immunkomplexbildung und Komplementaktivierung zu einer Kleingefäßvaskulitis führen kann^4^ [33, 34]. Klinisch findet sich eine MM oder sensomotorische PNP [32–34]. Einzelfallberichte einer VM sind ebenfalls für das SjS beschrieben worden [34].

Auch im Rahmen eines systemischen Lupus erythematodes kann sich in seltenen Fällen eine VN entwickeln [35–39].

## Einzelorganvaskulitis

Isolierte Vaskulitismanifestationen der Skelettmuskulatur und peripherer Nerven können als sogenannte „Einzelorganvaskulitis“ auftreten [40]. Die Entzündungsreaktion ist hierbei auf ein einzelnes Organ begrenzt, wenngleich der Organbefall sich mit fokalen oder diffusen Läsionen manifestieren kann [40]. Eine SOV der Skelettmuskulatur entwickelt sich typischerweise im Bereich der unteren Extremität und betrifft mittelgroße Arterien und Kleingefäße [41–44]. In sehr seltenen Fällen tritt eine SOV des peripheren Nervensystems typischerweise als lymphozytäre Vaskulitis kleiner und/oder mittelgroßer Arterien auf [40]. Die histopathologischen Befunde einer SOV sind dabei nicht von denen einer systemischen Vaskulitis abzugrenzen. Insgesamt sollte die Diagnose einer SOV zurückhaltend gestellt werden („Arbeitsdiagnose“) und die Erkrankten regelmäßig im Hinblick auf systemische Manifestationen untersucht werden.

## Vaskulitische Myopathie

### Klinische Perspektive.

**Abb. 2:**
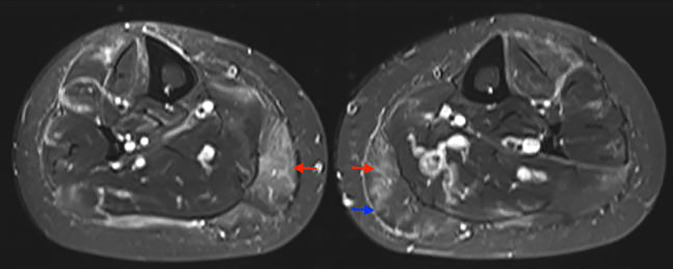
Magnetresonanztomografie der Unterschenkel beidseits (T2-Wichtung, axial): Nachweis von symmetrischen intramuskulären Ödemen (*rote Pfeile*) sowie einer Fasziitis (*blauer Pfeil*) im Rahmen einer Polyarteriitis nodosa. Histologisch konnte eine vaskulitische Myopathie durch den Nachweis einer nekrotisierenden Vaskulitis im Bereich kleiner epimysialer Arterien belegt werden

Aus klinischer Sicht können bei der VM neben immobilisierenden Myalgien auch eine Schwellung, Paresen und vegetative Symptome (z. B. Fieber) auftreten. Hierbei ist vorwiegend die untere Extremität distal betroffen^5^ [10, 11, 41, 45]. Zusätzlich sollte eine gezielte Evaluation hinsichtlich anderer Vaskulitismanifestationen erfolgen und insbesondere die Haut, das periphere Nervensystem und die Nierenfunktion umfassen. Typischerweise sind die serologischen Inflammationsparameter deutlich erhöht, während die CK-Aktivität in vielen Fällen normwertig (in Abhängigkeit von der Grunderkrankung sogar in bis zu 100 % der Fälle) oder nur leichtgradig erhöht ist [3, 10, 20]. Eine normwertige CK-Aktivität kann daher *nicht* zum Ausschluss einer vaskulitischen Myopathie herangezogen werden. Als wichtige Differenzialdiagnose sollte in diesem Zusammenhang auch eine Polymyalgia rheumatica bedacht werden. Zur weiteren Einordnung empfiehlt sich die Bestimmung von ANCA, RF, Anti-CCP-Antikörpern, antinukleären Antikörpern und Virusserologien (HBV, HCV, HIV). Bei differenzialdiagnostischer Unsicherheit (z. B. unklare Hautbefunde oder begleitende proximale Myalgien) kann die Bestimmung von Myositis-spezifischen bzw. -assoziierten Antikörpern sinnvoll sein [46]. Anschließend sollte eine Bildgebung^6^ der betroffenen Muskulatur erfolgen, vorzugsweise eine MRT. Von radiologischer Seite sind hierbei diffuse oder fleckige Hyperintensitäten in der T2-Wichtung verdächtig (Abb. 2; [10, 42, 43, 49, 50]). Im Gegensatz hierzu zeigen T1-Hyperintensitäten einen chronischen Prozess mit fibrotischem Umbau der Muskulatur an, sodass differenzialdiagnostisch an hereditäre Myopathien gedacht werden muss. Die Konstellation einer „MRT-Myositis ohne Myositis“ (MR-morphologischer Befund passend zu einer Myositis und normwertige CK) sollte zur Verdachtsdiagnose^7^ einer VM führen [8]. Die Indikation zur Muskelbiopsie ist abhängig von der klinischen Konstellation und sollte zur Diagnosesicherung erwogen werden, sofern dies nicht anders gelingt. Inwieweit zukünftig eine Diagnosestellung anhand bildgebender Verfahren (z. B. ^18^FDG-PET/CT) erfolgen kann, ist Gegenstand aktueller Forschung [12, 13]. Zur Therapie der vaskulitischen Myopathie (Tab. 3) sind Glukokortikoide (GK) indiziert, bei der PAN können ergänzend Methotrexat und Tocilizumab in refraktären Fällen („Off-label-Therapie“) [52, 53] eingesetzt werden. Sofern zusätzlich auch eine vaskulitische Neuropathie (VN) besteht, sollte mit CYC therapiert werden. Bei den AAV wird das therapeutische Vorgehen durch den weiteren Organbefall (organ- bzw. lebensbedrohender Verlauf) bestimmt (siehe aktuelle Empfehlungen der EULAR [54]).

**Tab. 3:** Therapeutische Optionen zur Remissionsinduktion bei vaskulitischer Myopathie und Neuropathie

Vaskulitis	Myopathie	Neuropathie
Polyarteriitis nodosa	GK + MTX ggf. Tocilizumab oder CYC	GK + CYC
ANCA-assoziierte Vaskulitis	GPA/MPA: GK + MTX oder GK + RTX EGPA: GK	GPA/MPA: GK + CYC oder GK + RTX EGPA: GK + CYC
Kryoglobulinämische Vaskulitis	GK ± RTX	
Rheumatoide Vaskulitis	GK + TNF oder RTX oder CYC	

Abkürzungen: *CYC* Cyclophosphamid, *EGPA* eosinophile Granulomatose mit Polyangiitis, *GPA* Granulomatose mit Polyangiitis, *GK* Glukokortikoide, *MPA* mikroskopische Polyangiitis, *RTX* Rituximab, *TNF* TNF-α-Antagonisten

### Histopathologische Begutachtung.

**Abb. 3:**
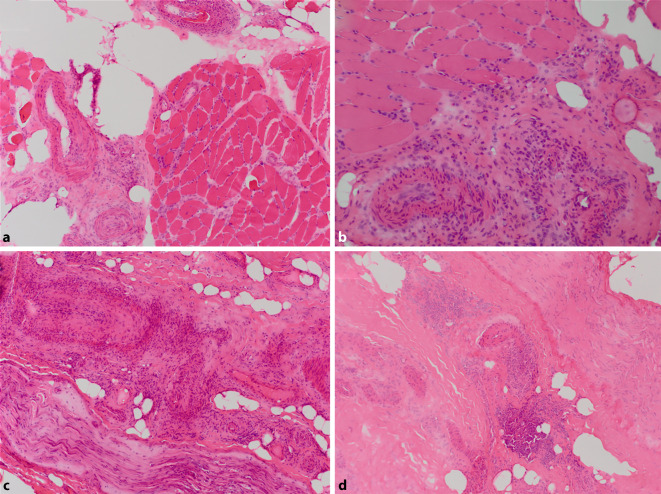
Histopathologische Befunde bei vaskulitischer Myopathie (**a**, **b**) und Neuropathie (**c**, **d**) im Rahmen unterschiedlicher Grunderkrankungen. **a** Entzündliche Infiltration kleiner epimysialer Arteriolen und Begleitinfiltrat in der angrenzenden Skelettmuskulatur bei rheumatoider Vaskulitis (Hämatoxylin-Eosin, x200). **b** Entzündliche Infiltration der Gefäßwand mit partieller Destruktion der Wandanteile bei eosinophiler Granulomatose mit Polyangiitis (Hämatoxylin-Eosin, x200). **c** Entzündliche Infiltration und Destruktion der Gefäßwand kleiner epineuraler Arterien bei rheumatoider Vaskulitis (Hämatoxylin-Eosin, x100, Nervenpräparat im Längsschnitt). **d** Histopathologischer Nachweis einer Kleingefäßvaskulitis im Bereich epineuraler Gefäße bei kryoglobulinämischer Vaskulitis (Hämatoxylin-Eosin, x100, Nervenpräparat im Längsschnitt)

Im Allgemeinen wird empfohlen, eine kombinierte Biopsie von Muskel und Nerv durchzuführen, um die diagnostische Trefferquote zu erhöhen [14, 15, 55, 56]. Es wird empfohlen, Kryomaterial für sämtliche Untersuchungen zu nutzen, wobei für einzelne immunhistochemische Parameter auch Paraffingewebe nützlich sein kann. Paraffingewebe hat jedoch den Vorteil, dass es unproblematischer gelagert werden kann, was in Settings außerhalb hoch spezialisierter Zentren ein Argument sein kann. Die Vaskulitis wird auf der histologischen Ebene über die Gefäßwandinfiltration von lymphomonozytären Zellelementen gesichert (Abb. 3a, b). Diese „Infiltration“ kann verschiedene Charakteristika aufweisen und variiert von „einfacher Durchwanderung“ bis zur Wanddestruktion. Auch Riesenzellen können im Falle der Sarkoidose in der Gefäßwand erkennbar sein. Der Nachweis von Granulozyten (z. B. Eosinophile) kann Hinweise auf den immunologischen Kontext geben (z. B. ANCA-Assoziation). Da in Nerven- und Muskelbiopsien lediglich kleine bis mittelgroße Gefäße angetroffen sind, ist eine Aussage auch nur über diese Kaliber möglich und eine direkte Vergleichbarkeit mit der Chapel-Hill-Nomenklatur, die nach Gefäßkaliber einordnet, nur sehr begrenzt möglich. Außerdem sollte eine Gefäßassoziation wie z. B. bei vielen adulten Formen der Dermatomyositis, wo es typischerweise zum Nachweis von lymphomonozytären Zellen in Nähe perimysialer Gefäße kommt, nicht mit einer primären Vaskulitis verwechselt werden. Andererseits darf die Hochregulation von MHC-Klasse-I-Molekülen (major histocompatibility complex) auf dem Sarkolemm von Muskelfasern perifokal bei Vaskulitis nicht zu der Fehldiagnose einer Dermatomyositis führen. Im Zweifel sind auch ultrastrukturelle Untersuchungen geeignet, um Klarheit zu schaffen, denn nur die Dermatomyositis zeigt tubuloretikuläre Inklusionen in Kapillaren.

## Vaskulitische Neuropathie

### Klinische Perspektive.

Die VN geht häufig mit Schmerzen, körperlichen Funktionseinschränkungen und einer reduzierten Lebensqualität einher [57], sodass die rechtzeitige Diagnose und Therapie von wesentlicher Bedeutung sind. Aus pathophysiologischer Sicht sollte das Auftreten einer peripheren Neuropathie im Rahmen einer SV oder anderen Grunderkrankung^8^ von einer SOV („nonsystemic vasculitic neuropathy“, NSVN) getrennt werden [59]. Die Grundlage hierfür bildet eine umfangreiche Organdiagnostik, welche vorwiegend die Lunge, Niere und Haut erfassen sollte. Umgekehrt sollte eine VN bei neuropathischen Beschwerden im Kontext einer bereits etablierten SV evaluiert werden. Anamnestisch werden Schmerzen, sensible Ausfall- (Hypästhesie, Anästhesie) und Reizerscheinungen (Kribbelparästhesien, brennende Schmerzen) sowie motorische Defizite berichtet [60]. Hierbei ist vorwiegend die untere Extremität betroffen. Hoch verdächtig sind periphere sensomotorische Defizite im Versorgungsgebiet eines peripheren Nervs mit akutem bis subakutem Verlauf [61] (nach Ausschluss einer traumatischen Genese) im Sinne einer Mononeuritis, die im Verlauf stufenweise weitere Nerven betreffen können (MM) [62]. In der klinisch-neurologischen Untersuchung lässt sich ein multifokales Verteilungsmuster mit distaler Betonung identifizieren, das am häufigsten den Nervus peroneus bzw. den Nervus ulnaris betrifft [60, 63]. Ein Befall der Hirnnerven tritt vergleichsweise selten auf, am ehesten bei der GPA. Typischerweise besteht eine MM (asymmetrisches Verteilungsmuster), die den Verdacht auf eine VN lenkt. In Abhängigkeit von der Grunderkrankung und der zeitlichen Dynamik kann jedoch auch das Bild einer distal symmetrischen PNP bestehen^9^. Die Bestimmung von Neurofilament-Leichtketten im Serum kann bei entsprechendem klinischen Kontext (AAV) auf eine aktive VN hindeuten [64]. Mithilfe elektrophysiologischer Diagnostik kann der klinische Verdacht weiter erhärtet und ein geeigneter Nerv für eine Biopsie ausgewählt werden [15, 62]. In der Nervenleitgeschwindigkeit zeigt sich typischerweise ein axonaler Schaden. Ferner gilt eine Amplitudendifferenz von ≥ 50 % im Seitenvergleich (asymmetrische Neuropathie) hierbei als diagnostisch bedeutsam [63]. Bildgebende Verfahren (Nervensonografie^10^ und MR-Neurografie^11^) können die Diagnostik von peripheren Neuropathien unterstützen [15] und die Lokalisation nervaler Strukturen für eine Nervenbiopsie erleichtern. Die Indikation zur Nervenbiopsie ergibt sich bei hinreichend schwerer oder progredienter Neuropathie, sofern eine ätiologische Zuordnung nicht anderweitig möglich ist und eine Behandlungskonsequenz (Immunsuppression) besteht [15]. Bei vorwiegend sensiblen Reizsymptomen (z. B. Brennen, Ameisenlaufen) und blander Elektrophysiologie sollte auch an das Vorliegen einer isolierten sog. „Small-fiber-Neuropathie“ (SFN)^12^ gedacht werden, welche kleinste, unmyelinisierte C‑Fasern betrifft. Hier kann eine (ergänzende) Hautbiopsie mit Bestimmung der intraepidermalen Nervenfaserdichte die Diagnose sichern. Zur Therapie der VN (Tab. 3) ist eine Kombinationstherapie mit GK und CYC indiziert (wie bei organbedrohendem Verlauf einer AAV [54]). Bei AAV, KV und RV kann Rituximab als Alternative zu CYC eingesetzt werden. Intravenöse Immunglobuline („Off-label-Therapie“) stellen eine Therapieoption bei refraktärem Verlauf einer AAV dar. Zur Therapie der mit einer VN verbundenen neuropathischen Schmerzen eignen sich Pregabalin und Gabapentin. Bei therapierefraktären Schmerzen haben sich auch Duloxetin, Amitriptylin und Natriumkanalblocker (z. B. Lacosamid) bewährt.

### Histopathologische Begutachtung.

Für die Untersuchung der peripheren Nerven wird häufig eine Biopsie des Nervus suralis (lediglich kleines sensibles Versorgungsgebiet) durchgeführt. Dies ist in den meisten Fällen ausreichend. Sollte klinisch ausschließlich eine Mononeuritis vorliegen, ist zu erwägen, ob der betroffene Nerv für die Diagnostik genutzt werden kann. Für die Materialasservierung gelten dieselben allgemeinen Bemerkungen wie für die Asservierung von Skelettmuskulatur. In jedem Falle erhöht auch hier die Kombination aus Haut‑, Nerven- und Muskelbiopsie die Trefferquote erheblich [14, 55, 56]. Analog zur Untersuchung der Skelettmuskulatur steht der Nachweis einer Gefäßwandinfiltration von lymphomonozytären Zellelementen im Vordergrund der histopathologischen Diagnosestellung (Abb. 3c, d).

## Fazit für die Praxis

- Das periphere Nervensystem und die Skelettmuskulatur sind häufige Zielorgane von systemischen Vaskulitiden. Myalgien, Paresen und Sensibilitätsstörungen sind in diesem Zusammenhang typische Krankheitszeichen.
- Eine vaskulitische Affektion der Skelettmuskulatur und peripherer Nerven tritt vorwiegend bei der Polyarteriitis nodosa und Kleingefäßvaskulitiden auf.
- Eine vaskulitische Neuropathie kann sich auch als Einzelorganvaskulitis manifestieren.
- Der Goldstandard zur Diagnosesicherung ist die histopathologische Untersuchung von Nerven- bzw. Muskelbiopsien mit Nachweis lymphomonozytärer Gefäßwandinfiltration.

## Footnotes

1 Alternativ gewinnt die Einteilung nach ANCA-Spezifität (im Gegensatz zur phänotypischen Kategorisierung in die drei o. g. Entitäten) zunehmend an Bedeutung, da sich beispielsweise der Krankheitsverlauf und das Therapieansprechen besser anhand des serologischen Status vorhersagen lassen.

2 Eine ältere Arbeit von Magyar et al. [30] untersuchte Muskelbiopsien von 100 Erkrankten mit RA. Interessanterweise zeigten sich hier vaskulitische Veränderungen im Bereich großer Arteriolen und kleiner Arterien.

3 Mertz et al. [30] haben alternative Diagnosekriterien für die RV vorgeschlagen, die nicht zwingend den histologischen Nachweis vaskulitischer Veränderungen fordern.

4 Aus pathophysiologischer Sicht sollten auch nicht-vaskulitische Prozesse (z. B. Ganglionitis der Hinterwurzel) in der Differenzialdiagnostik berücksichtigt werden.

5 Eine Affektion der proximalen Muskulatur (z. B. Musculus quadriceps femoris) ist für die untere Extremität ebenfalls beschrieben worden [20]. Inwieweit Unterschiede im Befallsmuster der Muskulatur zwischen verschiedenen Vaskulitiden bestehen, wurde bisher nicht untersucht.

6 Die Muskelsonografie stellt eine potenzielle Ergänzung dar, mit der Myopathien nichtinvasiv untersucht werden können [47]. Bei der Dermatomyositis ist beispielsweise eine vermehrte Echogenität der affektierten Muskulatur beschrieben worden [47]. Eine ältere Studie von Reimers et al. [48] untersuchte verschiedene Myositisentitäten und dokumentierte einen engen Zusammenhang zwischen den Ultraschallbefunden und dem Ergebnis der Muskelbiopsie. Für die vaskulitische Myopathie ist der diagnostische Nutzen der Muskelsonografie jedoch nicht untersucht worden.

7 Differenzialdiagnostisch ist auch eine subklinische Inflammation bei Dermatomyositis mit amyopathischem Verlauf zu bedenken [51].

8 In seltenen Fällen kann sich eine VN auch im Rahmen anderer Systemerkrankungen, z. B. bei Sarkoidose manifestieren [58].

9 Einige Autor:innen [62] beschreiben die distal symmetrische PNP sogar als die häufigste Manifestation der VN.

10 Die Nervensonografie gewinnt zunehmend an Bedeutung für die Diagnostik von peripheren Neuropathien [15, 65–67]. Hiermit können die Nervenquerschnittsfläche und Faszikelgröße bestimmt und Veränderungen der Echogenität dargestellt werden. Bisher ist der Stellenwert der Nervensonografie für die Diagnose der VN nur anhand von kleinen Kohorten untersucht worden [68–71]. Im Vergleich zu gesunden Kontrollen ist beispielsweise eine Vergrößerung der Nervenquerschnittsfläche bei VN beschrieben worden [68].

11 Als bildgebendes Verfahren wird die MR-Neurografie vermehrt in der Diagnostik extrakranieller Neuropathien eingesetzt [72] und hat auch Eingang in die S1-Leitlinie „Diagnostik bei Polyneuropathien“ [15] gefunden. Allerdings betont diese Leitlinie deutlich den Stellenwert der Nervenbiopsie bei Vaskulitiden. Für die VN ist der diagnostische Nutzen der MR-Neurografie bisher nur für die NSVN [73] untersucht worden und gegenwärtig nicht routinemäßig etabliert [74].

12 Es handelt sich nicht um ein primär entzündliches Krankheitsbild, weshalb eine immunsuppressive Therapie keine Anwendung findet. Vaskulitische Veränderungen lassen sich in Hautbiopsien nicht nachweisen.
